# Multivariate Radiological-Based Models for the Prediction of Future Knee Pain: Data from the OAI

**DOI:** 10.1155/2015/794141

**Published:** 2015-10-04

**Authors:** Jorge I. Galván-Tejada, José M. Celaya-Padilla, Victor Treviño, José G. Tamez-Peña

**Affiliations:** ^1^Grupo de Investigación en Bioinformática, Escuela de Medicina, Tecnológico de Monterrey, 64849 Monterrey, NL, Mexico; ^2^Departamento de Investigación e Innovación, Escuela de Medicina, Tecnológico de Monterrey, 64710 Monterrey, NL, Mexico

## Abstract

In this work, the potential of X-ray based multivariate prognostic models to predict the onset of chronic knee pain is presented. Using X-rays quantitative image assessments of joint-space-width (JSW) and paired semiquantitative central X-ray scores from the Osteoarthritis Initiative (OAI), a case-control study is presented. The pain assessments of the right knee at the baseline and the 60-month visits were used to screen for case/control subjects. Scores were analyzed at the time of pain incidence (T-0), the year prior incidence (T-1), and two years before pain incidence (T-2). Multivariate models were created by a cross validated elastic-net regularized generalized linear models feature selection tool. Univariate differences between cases and controls were reported by AUC, *C*-statistics, and ODDs ratios. Univariate analysis indicated that the medial osteophytes were significantly more prevalent in cases than controls: *C*-stat 0.62, 0.62, and 0.61, at T-0, T-1, and T-2, respectively. The multivariate JSW models significantly predicted pain: AUC = 0.695, 0.623, and 0.620, at T-0, T-1, and T-2, respectively. Semiquantitative multivariate models predicted paint with *C*-stat = 0.671, 0.648, and 0.645 at T-0, T-1, and T-2, respectively. Multivariate models derived from plain X-ray radiography assessments may be used to predict subjects that are at risk of developing knee pain.

## 1. Introduction

Knee pain is the most common and disabling symptom of Osteoarthritis (OA) [1, 2]. This disease affects 1 in every 10 adults over 60 years in the United States and the rate of incidence is incrementing due to changes in lifestyle and life expectancy [3–7]. The prevalence and the symptomatic importance of pain in OA subjects make pain prediction a very important task for the management of OA patients. Pain is a late manifestation of a pathological change in joint tissues; therefore, the early detection of pathological process may be used to determine who is at risk of developing OA related pain. This early detection of the underling pathology may be possible with the aid of noninvasive procedures like medical imaging. Medical imaging has proved to be a very important and effective tool in OA diagnosis; it is also the most common first-hand information for physicians and a probed form to obtain a good approach to OA staging [8–13].

Due to its maturity, simplicity and broad base deployment of X-Ray, it is the primary medical imaging modality used in OA diagnosis and staging. Radiological OA has been defined as subjects presenting bone alterations (osteophytes) and reduced joint space [14]. This findings have been correlated to joint symptoms of pain and stiffness [15]; but the bony changes prognosis power have not been properly studied in longitudinal studies [16–19]. The biggest challenge facing radiological correlation to symptomatic OA is the multifactorial source of joint pain and the subjective perception of pain [20]. Other challenge has been the lack of standardized image assessment procedures that allow a proper evaluation and comparisons of OA studies. To overcome these limitations, validated subject questionnaires [21, 22] and standardized image assessments have been developed [23–26].

The Osteoarthritis Initiative (OAI) has been recollecting thousands of clinical data in OA patients, subjects at risk, and control subjects using validated questionnaires and standardized image assessments procedures. The OAI effort brings very important information that will offer a better understanding of the disease process.

In this work, the OAI X-ray quantitative image assessments of joint space width, the central reads of Kellgren and Lawrence (K&L), and the Osteoarthritis Research Society International (OARSI) scores are explored in their association to concurrent and future knee pain. The number of radiological findings as reported by the OAI central image assessments is large: osteophytes, bone attrition, and reduced joint space at the medial and lateral aspects of the joint. The OAI quantitative image analysis of joint space also provides a set of measurements that makes data exploration a challenge. This large array of radiological features and its association to pain cannot be handled by simple statistical analysis tools. Advanced feature selection and bioinformatics tools provide a proven method to handle this complex issue [27–30]. These advanced methods automatically build simple multivariate models that best describe the association of radiological features with pain.

This work explores the use of this bioinformatics tools to build radiological multivariate models of future joint pain and concurrent joint pain, with the objective of finding what radiological features or models can be used in to determine which set of people with radiological OA findings is at greater risk developing knee pain.

This paper is organized as follows; after introduction, the patients selection and methods of selection are explained, in Data Acquisition, the process of image feature acquisition is presented; in Statistical Analysis, the complete transformations and data analysis are explained; in Results, the tables with the numerical results are presented; finally, Discussion, Conclusion, and the future work are presented.

## 2. Patients Methods

*Study Population*. “Data used in the preparation of this article were obtained from the Osteoarthritis Initiative (OAI) database, which is available for public access at http://www.oai.ucsf.edu/datarelease/.” All subjects were selected from OAI databases. Based on the available information, this study was designed for right knee only.

Being a pain prediction study, the development of chronic pain in the right knee was used as the variable to look at. Using the five-year screening information, a group of subjects was selected. All subjects should not present chronic pain as a symptom in their baseline visit and should not have been medicated for pain.

Control subjects were selected under the criteria of the following: not presenting pain as a symptom since the baseline visit to 60-month visit, not presenting a symptomatic status since the baseline visit to 60-month visit, and taking no pain medication from the baseline visit to the 60-month visit. Case subjects were selected under the criteria of the following: not presenting pain as a symptom at baseline visit, not presenting a symptomatic status at baseline visit, taking no pain medication at the baseline visit, and developing chronic right knee pain in some time point after baseline and up to 60-month visit.

**Figure 1:**
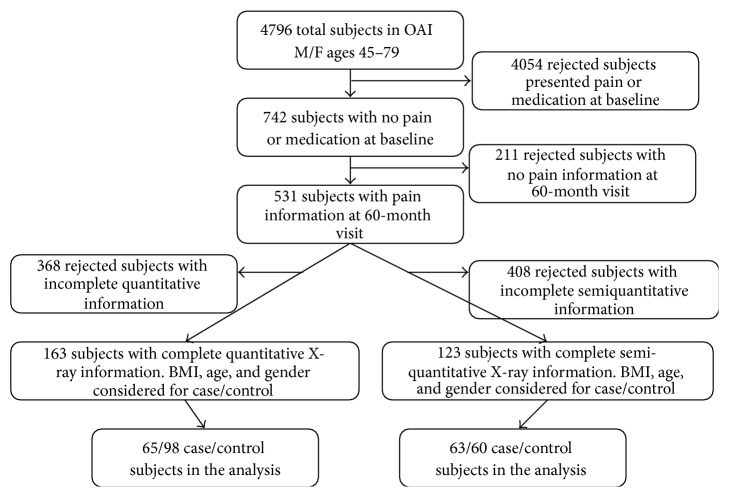
Subject selection.

Only the subjects with a complete quantitative or semiquantitative X-ray assessment screening were included in the final test. Due to this last condition, two different groups were created, one for quantitative study and one for semiquantitative study. All demographic information is presented in Table 1, and the selection process is described in detail in Figure 1.

## 3. Data Acquisition

In this analysis, right knee assessment from the OAI datasets, “central assessment of longitudinal knee X-rays for quantitative JSW” version 1.6, was right knee assessment from OAI dataset “central reading of knee X-rays for K-L grade and individual radiographic features of knee” version 1.6, and the outcome information was chronic pain, defined by the question in the OAI dataset “right knee symptom status.” This information was preanalyzed by two different radiologist groups associated with the OAI; one group evaluated the images using the OARSI quantitative grading scale [25, 31] and the semiquantitative K-L grading scale [26, 32, 33]. In Table 2, a description of the assessed features and their IDs are presented.

**Figure 2:**
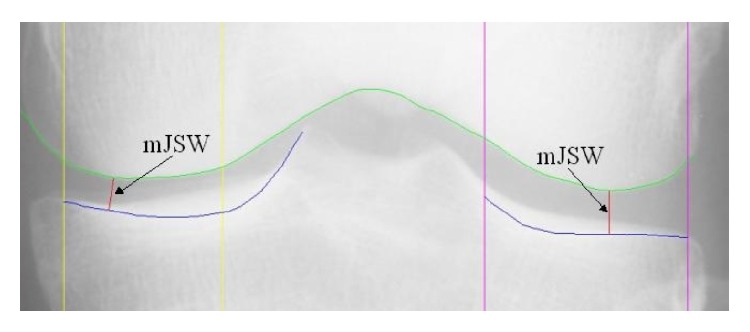
Output of the software on a digital knee radiograph. mJSW is the minimum JSW in a compartment. We provide mJSW for the medial compartment only (image from OAI).

All X-ray images were assessed using the OAI method; automated computational software and an external reader delineate the margin of the femoral condyle and the tibial plateau; in Figure 2, an example of the software output is presented.

**Figure 3:**
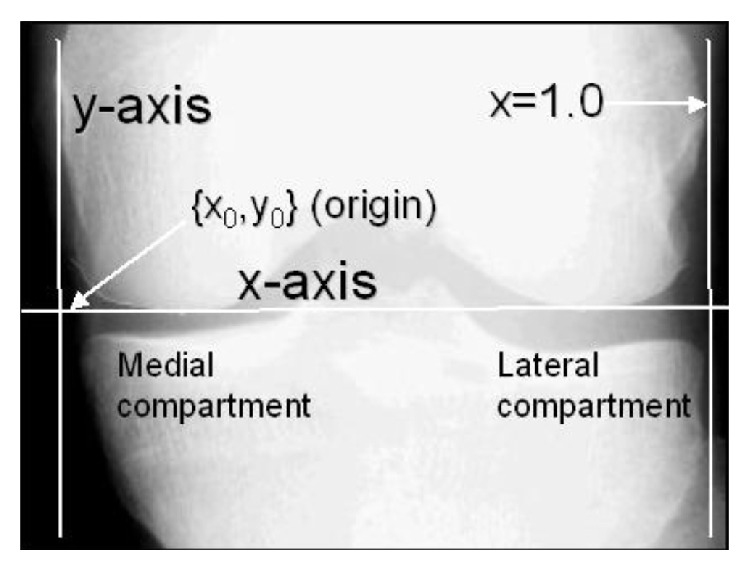
Landmarks and definition of coordinate system (image from OAI).

**Figure 4:**
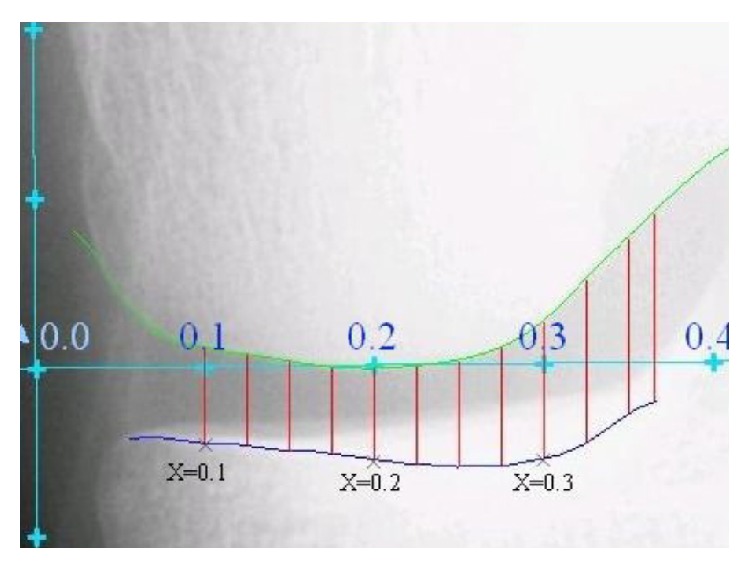
Measurement of JSW(*x*). Measurements from *x* = 0.150 mm to *x* = 0.300 mm are provided in increments of 0.025 mm (image from OAI).

Using an anatomical coordinate system, an objective *x*-location is determined. In Figure 3, an example of the reader line is presented. According to OAI information, a study of longitudinal knee radiographs suggested that *x* = 0.2 mm to *x* = 0.275 mm may be the optimal range for measuring medial JSW(*x*); an example of this measurements is presented in Figure 4.

All semiquantitative variables assessed for this work included the standard OAI protocol. This vendor includes Kellgren and Lawrence (K&L) grades, individual radiographic features (IRFs) such as osteophytes, and joint space narrowing in specific anatomic locations, based on published atlases.

In general, two expert readers independently assessed each film, blinded to each other's reading and to a subject's clinical data. Baseline and follow-up films were scored while being viewed simultaneously and with the readers blinded to chronological order of the images with the baseline film known and follow-up films randomly ordered.

## 4. Statistical Analysis

For quantitative and semiquantitative data, using the time of pain incidence as a marker, three different groups were built: T-0, using the radiological data of the subject at the moment of chronic pain development; T-1, using the radiological data on the subject a year before chronic pain development; and T-2, using the radiological data of the subject two years before chronic pain development. Seventeen quantitative variables and nineteen semiquantitative variables were explored in this work.

For the data analysis, the groups were analyzed using univariate and multivariate techniques. In both cases, for quantitative data, allometric association of joint height and gender to joint space width was adjusted using a linear regression [34], a common technique in related literature. All quantitative data was Z normalized using the rank inverse normal transform [35] using the standard levels of normalization reported in literature [36].

Seventeen quantitative features were measured in right knee assessments; the description of the features is shown in Table 2. To avoid the gender bias, all image features from the quantitative datasets went through a height and gender adjustment using a linear regression presented in the following:

(1) $$\begin{array}{c} \text{JSWadj}=\text{JSW}-b_{0}-\left(\text{Height}*b_{1}\right)-\left(\text{Gender}*b_{2}\right), \end{array}$$

where JSWadj represents the adjusted measurement, JSW is the original measurement, and *b*_0_, *b*_1_, and *b*_2_ are the coefficients obtained from the linear regression.

Due to the nature of the distribution of the binary outcome variable, in both cases (quantitative and semiquantitative), the univariate analysis was performed using logistic regression as a cost function using all features presented in Table 2. A general linear model, odds ratios, and the area under the Receiver Operating Characteristic (ROC) curve (AUC) were calculated on each feature; the ROC curve was constructed for each quantitative analysis, and the curve is a graphical representation of the sensitivity against* 1-specificity* for a binary classifier system as the discrimination threshold is varied.

Semiquantitative and quantitative data were analyzed independently. In both analyses, the different groups determined by the time of impact were tested independently to avoid bias.

In multivariate analysis, in order to select the best combination of features for the quantitative and semiquantitative prediction models, a multivariate search strategy was performed using elastic-net regularized generalized linear models as a classifier (LASSO) [37–39], with a* 10-fold* cross validation as a feature selection strategy; this method is commonly used in classification works. Accuracy, AUC, *C*-stat, and confusion matrix were obtained; in order to minimize the residual error of the prediction model, the lambda used in this research was chosen at lambda* = *lambda·min [37]. Lambda for quantitative was 0.037, 0.029, and 0.069, at T-0, T-1, and T-2, respectively. Lambda for semiquantitative was 0.062, 0.062, and 0.048, at T-0, T-1, and T-2, respectively.

All the statistical analysis was performed using R software and packages [40].

## 5. Results

The statistical description trough the time points of each quantitative and semi quantitative features are presented in Tables 3 and 4.

In the univariate analysis, all quantitative features showed not to be predictive by it self. In the semi-quantitative features, the “Osteophytes (OARSI grades 0–3) femur medial compartment (XROSFM)” showed to be predictive. In Tables 5 and 6 the complete statistical results of all the individual features are presented.

**Figure 5:**
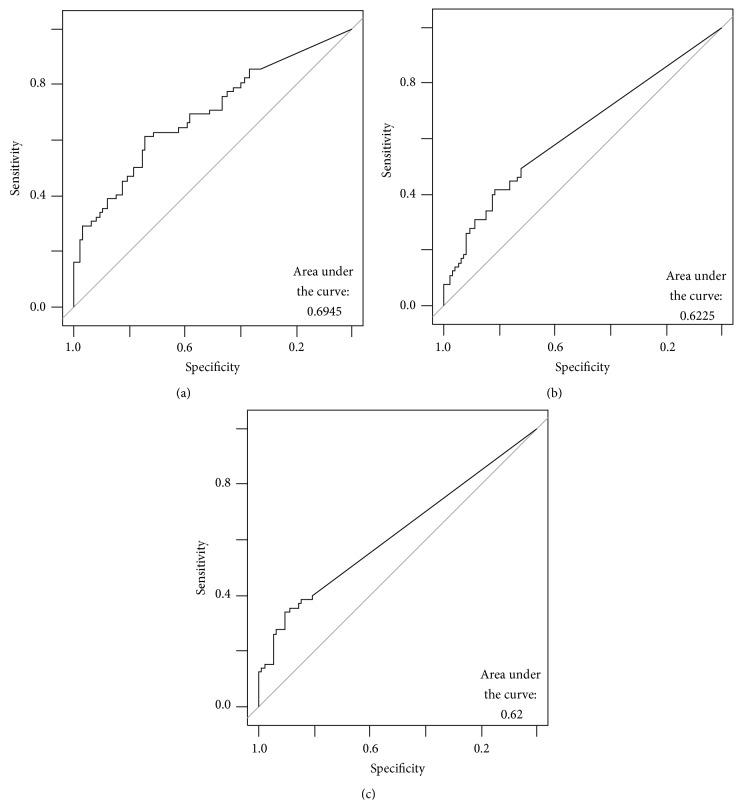
AUC curves on quantitative models: (a) curve for T-0, (b) curve for T-1, and (c) curve for T-2.

Using multivariate analysis of quantitative data three predictive models were obtained. For the time of pain incidence, a six features predictive model obtained the best accuracy and AUC. In the one year before pain incidence, a two feature predictive model obtained the best accuracy and AUC. In the two years before the pain incidence a two features predictive model obtained the best accuracy and AUC, the resulting curves are presented in Figure 5. In Table 7, a complete results and statistical analysis of each model is presented.

Using multivariate analysis on semi-quantitative data three predictive models were obtained. For the time of pain incidence, a four features predictive model obtained the best accuracy and* C*-stat. In the one year before pain incidence, a two feature predictive model obtained the best accuracy and* C*-stat. In the two years before the pain incidence a three features predictive model obtained the best accuracy and* C*-stat. In Table 8, a complete results and statistical analysis of each model is presented.

## 6. Discussion

This case-control longitudinal analysis of subjects with chronic right knee pain found an association between radiographic evidence of early OA changes and the future onset of chronic pain symptoms. Specifically, it was found that particular radiological changes in knee anatomy are present at least two years in advance of the onset of chronic pain for a selected group of patients. Therefore, these results may indicate that specific changes in joint space and bony structure are risk factors for the future development of OA related pain. These findings reinforce the conclusions of several population-based studies that have reported that persons with radiographic knee OA are at higher risk of pain development compared to persons without radiological OA [11–13, 16, 18, 41, 42]. The reported radiological features may be added to the well-known risk factors of OA severity like varus-valgus mal-alignment [43].

The quantitative driven multivariate predictive models presented in Table 7 may indicate an association between the medial cartilage abnormalities and the chronic pain. The presence of lateral and medial osteophytes in the semiquantitative multivariate models reported in Table 8 was associated with chronic pain development. The changes in the medial JSW (JSW *x* = 0.275 or *x* = 0.300), bony damage, and Chondrocalcinosis were present two years before the pain occurrence. The individual features (Tables 5 and 6) were not as predictive as the multivariate models as expected given the fact that OA is a whole organ disease that affects several tissues at the same time [44].

When comparing these results to our previous efforts [45, 46], we saw an increase in the AUC from 0.652, 0.617, and 0.674 to 0.695, 0.623, and 0.620, at T-0, T-1, and T-2 time points. Furthermore, the process will take less than 2 min of computation time contrasted to the 48 hrs of computation using the same machine. The models obtained using LASSO were more stable since the process is deterministic compared to the stochastic nature of the genetic algorithm of the original work.

There are several limitations to our study. First of all, pain is a subjective outcome that changes from person to person. Second, we limit the inclusion to subjects that were not taking pain medications; therefore, the number of those developed pains during the observation period was small. Third, we limit the exploration to right knee findings, and unilateral pain may be affected by the symptoms of the contra lateral knee. Given these limitations, we cannot generalize the findings and the external validation of the results is required to assess the clinical applicability of the models.

## 7. Conclusions

Even though pain is a very complex and subjective clinical outcome, the systematic analysis of objective radiological features was able to find a multivariate model that indicates that there are certain anatomical features that preceded the development of knee pain. A biomarker based on those features may be used to help physicians to choose the best therapy or course of action for patients that present those features. This represents a great area of impact especially in developing countries, where access to the high level of health care system is very restricted.

Based on these results, it is evident that multivariate models obtained by computational methods can make better use of radiological characteristics, increasing the chance for the future development of an effective computer assisted diagnosis and/or treatment selection system.

## Figures and Tables

**Table 1:** Demographic information.

	Quantitative analysis subjects			Semiquantitative analysis subjects		
	Cases	Controls	All	Cases	Controls	All
Subjects (females)	65 (38)	98 (55)	163 (93)	63 (35)	60 (26)	123 (61)
Average height (*σ*) [m]	1.69 (.09)	1.68 (.1)	1.68 (.09)	1.66 (.23)	1.69 (.1)	1.67 (.18)
Average BMI (*σ*)	27.05 (4.3)	26.27 (4)	26.58 (4.1)	27.27 (4.4)	28.48 (4.1)	27.86 (4.3)
Average age (*σ*)	62.69 (9.6)	61.80 (10.1)	62.15 (9.9)	65.02 (9.6)	66.72 (8.7)	65.85 (9.2)
Age range	46–78	45–79	45–79	46–78	47–79	46–79

**Table 2:** Features description.

Quantitative features		Semiquantitative features	
Feature ID	Description	Feature ID	Description
MCMJSW	Medial minimum JSW [mm]	XROSFM	Osteophytes (OARSI grades 0–3) femur medial compartment
JSW175	Medial JSW at *x* = 0.175 [mm]	XRSCFM	Sclerosis (OARSI grades 0–3) femur medial compartment
JSW200	Medial JSW at *x* = 0.200 [mm]	XRCYFM	Cysts (grades 0-1) femur medial compartment
JSW250	Medial JSW at *x* = 0.250 [mm]	XRJSM	Joint space narrowing (OARSI grades 0–3) medial compartment
JSW300	Medial JSW at *x* = 0.300 [mm]	XRCHM	Chondrocalcinosis (grades 0-1) medial compartment
JSW225	Medial JSW at *x* = 0.225 [mm]	XROSTM	Osteophytes (OARSI grades 0–3) tibia medial compartment
JSW150	Medial JSW at *x* = 0.150 [mm]	XRSCTM	Sclerosis (OARSI grades 0–3) tibia medial compartment
JSW275	Medial JSW at *x* = 0.275 [mm]	XRCYTM	Cysts (grades 0-1) tibia medial compartment
LJSW850	Lateral JSW at *x* = 0.850 [mm]	XRATTM	Attrition (OARSI grades 0–3) tibia medial compartment
LJSW900	Lateral JSW at *x* = 0.900 [mm]	XRKL	Kellgren and Lawrence (grades 0–4)
LJSW700	Lateral JSW at *x* = 0.700 [mm]	XROSFL	Osteophytes (OARSI grades 0–3) femur lateral compartment
LJSW825	Lateral JSW at *x* = 0.825 [mm]	XRSCFL	Sclerosis (OARSI grades 0–3) femur lateral compartment
LJSW750	Lateral JSW at *x* = 0.750 [mm]	XRCYFL	Cysts (grades 0-1) femur lateral compartment
LJSW875	Lateral JSW at *x* = 0.875 [mm]	XRJSL	Joint space narrowing (OARSI grades 0–3) lateral compartment
LJSW725	Lateral JSW at *x* = 0.725 [mm]	XRCHL	Chondrocalcinosis (grades 0-1) lateral compartment
LJSW800	Lateral JSW at *x* = 0.800 [mm]	XROSTL	Osteophytes (OARSI grades 0–3) tibia lateral compartment
LJSW775	Lateral JSW at *x* = 0.775 [mm]	XRSCTL	Sclerosis (OARSI grades 0–3) tibia lateral compartment
		XRCYTL	Cysts (grades 0-1) tibia lateral compartment
		XRATTL	Attrition (OARSI grades 0–3) tibia lateral compartment

**Table 3:** Quantitative data statistical information.

	T-0				T-1				T-2			
	Mean	S.D.	Max	Min	Mean	S.D	Max	Min	Mean	S.D.	Max	Min
MCMJSW	0.010	0.931	2.106	−3.389	0.041	0.885	2.468	−3.003	0.071	0.919	2.260	−2.723
JSW175	0.005	0.961	3.652	−3.704	0.052	0.922	3.745	−3.154	0.064	0.920	3.963	−2.484
JSW200	0.011	0.986	3.547	−3.908	0.064	0.957	3.422	−3.657	0.068	0.923	3.660	−2.805
JSW250	0.006	1.021	3.586	−4.014	0.055	0.983	3.359	−3.578	0.092	0.956	3.657	−2.698
JSW300	0.061	1.067	4.123	−2.410	0.054	1.089	4.014	−2.520	0.104	1.022	4.037	−2.022
JSW225	0.008	1.008	3.355	−4.175	0.062	0.966	3.225	−3.818	0.073	0.925	3.451	−2.958
JSW150	0.019	0.958	3.744	−3.730	0.041	0.913	3.709	−3.184	0.098	0.920	3.914	−2.523
JSW275	0.032	1.040	3.834	−3.548	0.049	1.030	3.833	−3.368	0.127	0.970	3.745	−2.131
LJSW850	0.059	1.280	3.393	−4.560	0.081	1.202	3.231	−3.934	0.061	1.117	2.799	−3.958
LJSW900	0.074	1.348	3.477	−4.789	0.093	1.247	3.652	−3.825	0.055	1.206	2.697	−3.960
LJSW700	0.004	1.560	4.453	−4.415	0.094	1.516	4.533	−4.244	0.134	1.451	4.522	−5.466
LJSW825	0.054	1.287	3.332	−4.672	0.091	1.188	3.380	−3.484	0.087	1.121	3.183	−3.393
LJSW750	0.025	1.387	4.000	−5.452	0.100	1.284	3.524	−3.407	0.128	1.258	3.868	−3.921
LJSW875	0.065	1.282	3.324	−4.645	0.066	1.192	3.040	−3.982	0.035	1.135	2.488	−4.237
LJSW725	0.016	1.433	4.446	−5.325	0.104	1.351	3.798	−3.464	0.188	1.426	4.082	−5.838
LJSW775	0.030	1.320	3.695	−4.949	0.121	1.228	3.406	−3.048	0.114	1.189	3.316	−3.001
LJSW800	0.040	1.298	3.555	−4.513	0.120	1.201	3.498	−3.070	0.099	1.136	3.091	−3.077

**Table 4:** Semiquantitative data statistical information.

	T-0				T-1				T-2			
	0	1	2	3	0	1	2	3	0	1	2	3
XROSFM	80	31	5	7	81	29	5	8	82	29	5	7
XRSCFM	97	17	9	0	98	16	9	0	98	18	7	0
XRCYFM	122	1	0	0	122	1	0	0	122	1	0	0
XRJSM	66	40	16	1	66	42	15	0	68	41	14	0
XRCHM	121	2	0	0	120	3	0	0	121	2	0	0
XROSTM	50	62	9	2	51	61	9	2	51	62	8	2
XRSCTM	97	13	13	0	97	14	12	0	101	10	12	0
XRCYTM	119	4	0	0	119	4	0	0	119	4	0	0
XRATTM	123	0	0	0	123	0	0	0	123	0	0	0
XRKL	18	12	69	21	19	15	69	19	21	16	67	18
XROSFL	85	30	4	4	89	27	3	4	88	29	3	3
XRSCFL	115	3	2	3	116	4	2	1	117	3	2	1
XRCYFL	122	1	0	0	122	1	0	0	122	1	0	0
XRJSL	111	5	3	3	113	5	3	2	114	4	4	1
XRCHL	121	2	0	0	121	2	0	0	121	2	0	0
XROSTL	84	30	7	2	88	27	6	2	91	26	4	2
XRSCTL	115	1	5	2	117	1	4	1	117	2	3	1
XRCYTL	118	5	0	0	120	3	0	0	120	3	0	0
XRATTL	121	2	0	0	121	2	0	0	121	2	0	0

**Table 5:** Univariate quantitative statistical results.

	T-0					T-1					T-2				
	*P*	AUC	OR	2.50%	97.50%	*P*	AUC	OR	2.50%	97.50%	*P*	AUC	OR	2.50%	97.50%
MCMJSW	0.84	0.48	1.23	0.87	1.75	0.99	0.50	1.00	0.70	1.43	0.27	0.46	1.23	0.87	1.75
JSW175	0.93	0.50	1.26	0.89	1.80	0.61	0.49	1.09	0.78	1.54	0.20	0.55	1.26	0.89	1.80
JSW200	0.83	0.51	1.23	0.88	1.76	0.54	0.51	1.11	0.80	1.55	0.23	0.54	1.23	0.88	1.76
JSW250	0.89	0.50	1.39	1.00	1.98	0.54	0.52	1.10	0.80	1.53	0.06	0.57	1.39	1.00	1.98
JSW300	0.34	0.53	1.43	1.05	2.00	0.18	0.55	1.22	0.91	1.65	0.03	0.58	1.43	1.05	2.00
JSW225	0.87	0.50	1.28	0.91	1.82	0.55	0.52	1.11	0.80	1.54	0.17	0.55	1.28	0.91	1.82
JSW150	0.74	0.52	1.25	0.89	1.79	0.69	0.51	1.07	0.76	1.52	0.20	0.55	1.25	0.89	1.79
JSW275	0.58	0.49	1.49	1.07	2.12	0.32	0.54	1.17	0.86	1.60	0.02	0.59	1.49	1.07	2.12
LJSW850	0.45	0.58	1.18	0.89	1.59	0.36	0.57	1.13	0.87	1.49	0.27	0.58	1.18	0.89	1.59
LJSW900	0.39	0.60	1.17	0.90	1.54	0.23	0.58	1.17	0.91	1.52	0.25	0.57	1.17	0.90	1.54
LJSW700	0.96	0.52	1.17	0.94	1.48	0.54	0.55	1.07	0.87	1.32	0.18	0.56	1.17	0.94	1.48
LJSW825	0.50	0.58	1.20	0.90	1.61	0.37	0.57	1.13	0.87	1.49	0.23	0.57	1.20	0.90	1.61
LJSW750	0.81	0.54	1.18	0.92	1.54	0.45	0.56	1.10	0.86	1.41	0.21	0.58	1.18	0.92	1.54
LJSW875	0.42	0.59	1.17	0.88	1.56	0.30	0.57	1.15	0.89	1.52	0.30	0.57	1.17	0.88	1.56
LJSW725	0.88	0.53	1.17	0.92	1.50	0.49	0.54	1.09	0.86	1.38	0.22	0.57	1.25	1.00	1.60
LJSW775	0.74	0.55	1.20	0.92	1.58	0.33	0.57	1.14	0.88	1.48	0.21	0.57	1.20	0.92	1.58
LJSW800	0.63	0.56	1.20	0.91	1.60	0.31	0.57	1.15	0.88	1.51	0.22	0.58	1.20	0.91	1.60

**Table 6:** Univariate semiquantitative statistical results.

	T-0					T-1					T-2				
	*P*	*C*-stat	OR	2.50%	97.50%	*P*	*C*-stat	OR	2.50%	97.50%	*P*	*C*-stat	OR	2.50%	97.50%
XROSFM	0.01	0.62	2.11	1.28	3.81	0.01	0.62	2.06	1.27	3.64	0.01	0.61	1.97	1.21	3.51
XRSCFM	0.22	0.53	1.48	0.80	2.85	0.28	0.53	1.41	0.77	2.72	0.40	0.52	1.33	0.70	2.63
XRCYFM	0.99	0.51	N/A	N/A	N/A	0.99	0.51	N/A	N/A	N/A	0.99	0.51	N/A	N/A	N/A
XRJSM	0.72	0.50	1.09	0.68	1.76	0.98	0.49	1.01	0.61	1.66	0.69	0.53	0.90	0.54	1.50
XRCHM	0.99	0.52	N/A	N/A	N/A	0.99	0.52	N/A	N/A	N/A	0.99	0.52	N/A	N/A	N/A
XROSTM	0.78	0.53	0.93	0.54	1.57	0.68	0.53	0.90	0.53	1.52	0.78	0.53	0.93	0.54	1.58
XRSCTM	0.78	0.50	1.08	0.63	1.88	0.88	0.50	1.04	0.60	1.84	0.91	0.52	0.97	0.55	1.71
XRCYTM	0.96	0.50	0.95	0.11	8.14	0.96	0.50	0.95	0.11	8.14	0.96	0.50	0.95	0.11	8.14
XRATTM	0.79	0.50	N/A	N/A	N/A	0.79	0.50	N/A	N/A	N/A	0.79	0.50	N/A	N/A	N/A
XRKL	0.07	0.58	1.42	0.98	2.12	0.39	0.53	1.18	0.81	1.75	0.78	0.51	1.05	0.72	1.54
XROSFL	0.02	0.59	2.00	1.14	3.82	0.08	0.56	1.68	0.97	3.14	0.05	0.57	1.89	1.05	3.73
XRSCFL	0.23	0.52	1.62	0.80	4.33	0.55	0.51	1.33	0.54	3.99	0.69	0.50	1.21	0.48	3.53
XRCYFL	0.99	0.51	N/A	N/A	N/A	0.99	0.51	N/A	N/A	N/A	0.99	0.51	N/A	N/A	N/A
XRJSL	0.23	0.53	1.48	0.82	3.10	0.62	0.51	1.20	0.59	2.65	0.62	0.51	1.22	0.57	2.88
XRCHL	0.99	0.52	N/A	N/A	N/A	0.99	0.52	N/A	N/A	N/A	0.99	0.52	N/A	N/A	N/A
XROSTL	0.05	0.57	1.77	1.02	3.27	0.18	0.54	1.48	0.85	2.71	0.20	0.53	1.49	0.83	2.86
XRSCTL	0.18	0.53	1.70	0.85	4.43	0.47	0.52	1.37	0.61	3.70	0.57	0.51	1.29	0.55	3.63
XRCYTL	0.22	0.52	4.00	0.57	79.50	0.59	0.51	1.93	0.18	42.28	0.59	0.51	1.93	0.18	42.28
XRATTL	0.99	0.52	N/A	N/A	N/A	0.99	0.52	N/A	N/A	N/A	0.99	0.52	N/A	N/A	N/A

**Table 7:** Multivariate models on quantitative X-ray data.

Time point	T-0			T-1			T-2		
Model variables	Medial JSW at *x* = 0.250 [mm]			Medial JSW at *x* = 0.275 [mm]			Medial JSW at *x* = 0.300 [mm]		
	Lateral JSW at *x* = 0.700 [mm]			Lateral JSW at *x* = 0.875 [mm]			Medial JSW at *x* = 0.275 [mm]		
	Lateral JSW at *x* = 0.750 [mm]								
	Lateral JSW at *x* = 0.800 [mm]								
	Medial JSW at *x* = 0.275 [mm]								
	Lateral JSW at *x* = 0.875 [mm]								

Accuracy	Accuracy: 0.688			Accuracy: 0.626			Accuracy: 0.632		

C.I.	95% CI: (0.6096, 0.7583)			95% CI: (0.5467, 0.7002)			95% CI: (0.5529, 0.706)		

AUC	0.695			0.623			0.620		

Confusion matrix	Pain			Pain			Pain		
	Pred	1	0	Pred	0	1	Pred	0	1
	0	91	43	0	94	57	0	92	54
	1	7	19	1	4	8	1	6	11

**Table 8:** Multivariate models on semiquantitative X-ray data.

Time point	T-0			T-1			T-2		
Model variables	Osteophytes (OARSI grades 0–3) femur medial compartment			Osteophytes (OARSI grades 0–3) femur medial compartment			Osteophytes (OARSI grades 0–3) femur medial compartment		
	Chondrocalcinosis (grades 0-1) medial compartment			Chondrocalcinosis (grades 0-1) medial compartment			Chondrocalcinosis (grades 0-1) medial compartment		
	Osteophytes (OARSI grades 0–3) femur lateral compartment						Osteophytes (OARSI grades 0–3) femur lateral compartment		
	Osteophytes (OARSI grades 0–3) tibia lateral compartment								

Accuracy	Accuracy: 0.6423			Accuracy: 0.626			Accuracy: 0.618		

C.I.	95% CI: (0.5509, 0.7267)			95% CI: (0.5342, 0.7116)			95% CI: (0.5259, 0.704)		

*C*-stat	0.671			0.648			0.645		

Confusion matrix	PAIN			PAIN			PAIN		
	Pred	0	1	Pred	0	1	Pred	0	1
	0	45	29	0	46	32	0	46	33
	1	15	34	1	14	31	1	14	30
